# Psychotic symptoms and its associated factors relating to psychoactive substance use among the youth population in Northwest Ethiopia

**DOI:** 10.3389/fpsyt.2023.1045111

**Published:** 2023-05-05

**Authors:** Tilahun Kassew, Sewbesew Yitayih Tilahun, Biruk Fanta Alemayehu, Berhanie Getnet, Demeke Demilew, Gebrekidan Ewnetu Tarekegn, Kassahun Alemu, Yassin Mohammed Yesuf, Mohammed Oumer, Eden Abetu Mehari, Mamaru Melkam, Goshu Nenko

**Affiliations:** ^1^Department of Psychiatry, College of Medicine and Health Sciences, University of Gondar, Gondar, Ethiopia; ^2^Department of Epidemiology and Biostatistics, Institute of Public Health, College of Medicine and Health Sciences, University of Gondar, Gondar, Ethiopia; ^3^Department of Psychology, College of Social Science and Humanities, University of Gondar, Gondar, Ethiopia; ^4^Department of Human Anatomy, School of Medicine, College of Medicine and Health Sciences, University of Gondar, Gondar, Ethiopia; ^5^Department of Clinical Pharmacy, School of Pharmacy, College of Medicine and Health, Sciences, University of Gondar, Gondar, Ethiopia

**Keywords:** psychoactive substances, youth, psychotic symptoms, Ethiopia, mental health

## Abstract

**Background:**

Substance use is associated with high rates of psychiatric symptoms including psychotic symptoms. Despite the severity of the problem, there are intervention gaps in Ethiopia. To combat this, relevant evidence is required to raise the awareness of service providers. This study aimed to assess the prevalence of psychotic symptoms and its associated factors among the youth population who uses psychoactive substances in the Central Gondar Zone, Northwest Ethiopia.

**Methods:**

A community-based cross-sectional study was conducted among the youth population in the Central Gondar zone, Northwest Ethiopia, from 01 January to 30 March 2021. A multistage sampling technique was used to recruit the study participants. All data were collected using questionnaires assessing socio-demographic; family-related variables; Depression, Anxiety, and Stress Scale; Multidimensional Scale Perceived Social Support (MSPSS), and Self-Reporting Questionnaire (SRQ-24). The data were analyzed using the STATA 14 statistical program.

**Results:**

A total of 372 young people who used psychoactive substances (79.57%, 53.49%, 34.14%, and 16.13% were consuming alcohol, Khat, tobacco/cigarette products, and other substances such as shisha, inhalants, and drugs, respectively) were included in the study. The prevalence of psychotic symptoms was 24.2% with a 95% CI: 20.1, 28.8. Being married [AOR = 1.87 95% CI: 1.06, 3.48], recent loss of loved ones [AOR = 1.97 95% CI: 1.10, 3.18], low perceived social support [AOR = 1.61 95% CI: 1.11, 3.02], and severe psychological distress [AOR = 3.23 95% CI; 1.64, 6.54] were the factors associated with psychotic symptoms among young people with psychoactive substances use at a *p*-value of < 0.05.

**Conclusion:**

Psychotic symptoms related to psychoactive substances among the youth population in Northwest Ethiopia were high. Thus, it is better to give a special attention to the youth population with low social support and existing psychological distress concurrent with psychoactive substance use.

## Introduction

Psychoactive substance use has become one of the major public health issues with its pervasive prevalence widespread across all categories of society (1, 2). According to the global addiction 2017 report, approximately 1 in 5 to 1 in 20 individuals aged 15 years reported using frequent consumption of alcohol, tobacco, and illicit drugs daily in the past month (3). Substance-related problems also cause significant economic impacts due to issues such as lost productivity and lives and healthcare costs (4). In general, substance use is more prevalent among young people. Adolescence is the beginning of the important period for initiation and young age, with the age between 18 and 25 being the beginning of the peak age for substance use (5, 6). Alcohol, tobacco, and cannabis have remained the substances that are most commonly used by young people around the world (4, 7). While tobacco is linked to the highest mortality, alcohol is the drug that is most frequently abused (8). According to a systematic review, in Eastern Africa, the prevalence of ever and problematic alcohol consumption was reported to be 52 and 15%, respectively (9).

The experiences of persecutory delusions, visual hallucinations, impulsivity and psychomotor agitation, affective and negative symptoms, a pervasive sense of unreality, and social and emotional issues are psychotic symptoms related to substance use (10). Numerous studies have found that young people who use psychoactive substances are more likely than the general population to experience psychotic symptoms (11, 12). Even though there are limited studies, psychotic-like experiences are prevalent in Ethiopia where the young group is highly exposed to the consumption of different psychoactive substances (13).

Users of psychoactive substances are known to experience psychotic symptoms, which are known to contribute to physical and psychological difficulties and have a variety of negative consequences on overall health outcomes (14). It can increase the likelihood of sexually transmitted diseases, accidental injuries, overdoses, and other illnesses such as lung, heart, and liver problems. In addition, it significantly increases the risk of behavioral disorders, such as violence and suicide, among young people. Psychotic symptoms also co-occur and worsen the progression of other mental disorders, such as depression, bipolar disorder, attention-deficit/ hyperactivity disorder, borderline personality, and antisocial personality disorder (15–17). Researchers reported that early adulthood, male gender, lower educational level, a lack of knowledge about the effects of alcohol use, having a husband who consumes alcohol, having partners and friends who consume alcohol and other substances, being unemployed, and people with low socioeconomic position were recognized as the main risk factors for the experiences of psychotic symptoms (11, 18, 19). The presence of mental problems, psychological distress, a lack of social support, the use of many drugs, and drug use for a longer period of time are all significant predictors of psychotic symptoms in young people (20–22).

Previous epidemiological studies did not pay much attention to the comorbidities of psychotic symptoms; instead, they focused on the prevalence of psychoactive substances in the juvenile population. Even though the impacts of psychotic symptoms on the health of youth people are frequent, there are limited studies conducted on psychotic symptoms among young people with substance use, in general, in Africa, specifically in Ethiopia, in particular. In addition, the young population has been using more psychoactive drugs recently, including alcohol, Khat, cigarettes, shisha, inhalants, and other drugs. The result of this study can deliver vital health information, which is crucial for policymakers to evaluate programs, design interventions, and strengthen the application of existing policies to treat and prevent psychotic-like experiences among substance use individuals who are aged between 15 and 25 years. To address the above gaps, we conducted this study with sufficient sample size and appropriate statistical analysis. Therefore, this study aimed to examine the prevalence and determinant factors of comorbid psychotic symptoms among the youth population with psychoactive substance use in Northwest Ethiopia.

### Hypothesis

- Do psychotic symptoms are high among the Northwest Ethiopian young people with psychoactive substance use?
- Do socio-demographic and psychosocial factors are the determinant factors for psychotic symptom experiences?

## Methodology of the study

### Study setting and population

A community-based cross-sectional study was carried out in the Central Gondar Zone of Northwest Ethiopia from 1 January to 30 March 2021. In Ethiopia, there are various administrative regions. Administratively, each region is divided into zones and zones into districts, which is the third administrative division of Ethiopia. Finally, in the fourth level, districts are further subdivided into Kebeles, the lowest administrative unit. Amhara regional state is one of the administrative regions, and the central Gondar zone is located in the northwest of the country. In this *zone*, there are 18 *districts* (17 rural *districts* and *Gondar special district*) and 442 *Kebeles* in total. The youth population is highly exposed to the use of different psychoactive substances in recent times.

The study participants were all individuals aged between 15 and 25 years who resided in the Central Gondar zone and used at least one psychoactive substance, such as alcohol, Khat, cigarettes and/or tobacco products, or other substances. The youth population who were unable to communicate because of a severe physical or mental illness during the time of data collection, however, were not included in the study. The initial objective's minimal sample size was calculated using a single population proportion calculation. Given that we used a multistage sampling technique, a design effect was taken into account while determining the sample size. The projected prevalence is based on a prior study (34.6%) conducted in northeast Ethiopia on high school students' usage of psychoactive substances (23). The calculated number was multiplied by two (2) for design effect as we go down two stages. Finally, the minimum required sample was 1,195 after adding a 10% non-response. However, we interviewed 1,396 young people. From the total, 372 individuals were consuming at least one psychoactive substance. Subsequently, we have undertaken a further assessment of the experiences of concurrent psychotic-like symptoms.

Since the study area is broad, a multistage sampling technique was employed to recruit the participants. We stand in the central Gondar zone (*Zonal level*) followed by the geographical cluster of each district and selected six districts. The number of participants in each randomly selected district was allocated proportionally based on the size of the youth population and then down to Kebele. The number of “Kebeles” are also randomly selected from these districts. There are also special centers “*Got*” inside Kebele. Here, we have recruited our participants from the *Got* in the form of a cluster by considering a proportionally allocated sample in each Kebeles. Initially, 1,396 young people were recruited to assess the prevalence of substance use. From the total, 372 participants were further interviewed for the assessment of experiences of concurrent psychotic-like symptoms. Therefore, for the objective of this study, further analysis was performed to determine the prevalence and determinants of psychotic symptoms among respondents with substance use.

### Data collection tools and procedures

The data were collected using standardized and structured questionnaires. The information from the study participants was gathered using a questionnaire that was completed by a face-to-face interview. The questionnaires were developed by adopting standardized tools and reviewing previous literature studies (24–27). The questionnaire was initially written in English before being translated into the indigenous Amharic tongue by bilingual translators. The questionnaire was retranslated into its original language to ensure compatibility with its English version. The questionnaire has four parts: (1) socio-demographic information and family-related variables (sex, age, marital status, religion, level of education, occupation, and present living situation; recent death of loved ones); (2) the Multidimensional Scale Perceived Social Support (MSPSS) tool; (3) the stress subscale of the Depression, Anxiety, and Stress Scale; and (4) the Self-Report Questionnaire (SRQ-24) psychotic symptoms.

### Instruments (tools)

#### Socio-demographic questionnaire

Data for basic socio-demographic and family-related characteristics of participants were collected using the socio-demographic and family information questionnaire prepared by the research team (24–27).

#### Self-reporting questionnaire (SRQ-24)

The outcome variable (psychotic symptoms) was assessed by using the SRQ-24 tool. SRQ-24 is an instrument with 24 items, which question respondents about symptoms and problems, 20 related to neurotic symptoms, and four items concerning psychotic symptoms. This study is interested to use SRQ-4 consisting only of the “psychotic” items for the assessment of psychotic symptoms at the community level. Each of the four items is scored 0 or 1. A score of 1 indicates that the symptom was present during the past 30 days; a score of 0 indicates that the symptom was absent. The maximum score is 4. Individuals with a total sum score of 2 or more are considered as having psychotic symptoms (28). Reliability analysis for the translated version was performed, and its Cronbach α value was 0.76.

#### Depression, anxiety, and stress scale-21 items

The Depression, Anxiety, and Stress Scale-21 Items (DASS-21) is a set of three self-report subscales designed to measure the emotional states of depression, anxiety, and stress. Each of the three DASS-21 scales contains seven items, divided into subscales with similar content. In this study, the stress subscale was used to measure the psychological stress level of the participants. The stress subscale is sensitive to levels of chronic non-specific arousal. It assesses difficulty in relaxing, nervous arousal, and being easily upset/agitated, irritable/over-reactive, and impatient. Scores for stress are calculated by summing the scores for the relevant items (29).

The responses for each statement scored on a Likert scale from 0 to 3 indicate how much the statement applied to you over the past week. The rating scales are “0,” Did not apply to me at all; “1,” Applied to me to some degree, or some of the time; “2,” Applied to me to a considerable degree or a good part of the time; and “3,” Applied to me very much or most of the time. Scores on the DASS-21 were needed to be multiplied by 2 to calculate the final score. The recommended cut-off scores for conventional severity labels for stress subscale (normal, mild moderate, and severe) are 0–14, 15–18, 18–25, and 26 and above considered as normal, mild, moderate, and severe, respectively. Each subscale items interclass correlation test was good with the Cronbach α value of 0.79, 0.78, and 85 for depression, anxiety, and stress scales, respectively. The DASS-21, therefore, has no direct implications for the allocation of patients to discrete diagnostic categories postulated in classificatory systems such as the DSM and ICD.

#### Multidimensional scale of perceived social support

The MSPSS is a 12-item scale designed to measure perceived social support from three sources: family, friends, and a significant other. The scale comprised a total of 12 items, with four items for each subscale (30). Each item's response is a Likert scale from 1 (very strongly disagree) to 7 (very strongly agree). The mean score for subscales is calculated as follows:

Significant other subscale: sum across items 1, 2, 5, and 10, and then divided by 4. Family subscale: sum across items 3, 4, 8, and 11, and then divided by 4. Friends subscale: sum across items 6, 7, 9, and 12, and then divided by 4. Total scale: sum across all 12 items, and then divided by 12. In this approach, any mean scale score ranging from 1 to 2.9 could be considered low support; a score of 3 to 5 could be considered moderate support; a score from 5.1 to 7 could be considered high support. Across many studies, the MSPSS has been shown to have good internal and test–retest reliability, good validity, and a fairly stable factorial structure (30, 31). In this study, the items interclass correlation test was good with its Cronbach α value of 0.81.

### Operational definitions

#### Youth

Youth is the young population denoted as the late adolescent and young adults aged between 15 and 24 years (35).

#### Psychoactive substance use

In this study, psychoactive substance use constitutes the use of at least one of the specified substances, such as alcohol, Khat tobacco/cigarette products, and other substances (shisha, inhalants, and drugs), either occasionally or daily based on the World Health Organization (WHO) Assessment of ASSIST tool (32).

#### Psychotic symptoms

Based on SRQ-24 psychotic items, individuals who scored 2 or more of the total sum score are considered having psychotic symptoms (28).

#### Perceived social support

The total mean score across all MSPSS-12 items, any mean scale score ranging from 1 to 2.9 could be considered low support; a score of 3 to 5 could be considered moderate support; a score from 5.1 to 7 could be considered high support (30).

#### Psychological stress

Based on the stress subscale of DASS, individuals with the total score of 0–14, 15–18, 18–25, and 26 and above are considered normal, mild, moderate, and severe, respectively (29).

### Data analysis procedure

EpiData 4.6 was used for statistical data entry, and STATA 14 was used for analysis after the data had been exported. The distribution of the data was summarized using descriptive statistics including frequency, percent, mean, and standard deviation. Binary logistic regression was employed to identify the associated factors with psychotic symptoms. In the bi-variable analysis, statistically significant variables using a cut-off *p*-value of < 0.25 were selected to identify candidate variables for multivariable analysis. Adjusted odds ratio with a 95% confidence interval was used to declare statistically significant variables based on a *p*-value of < 0.05 in the multivariable logistic regression model.

### Ethics approval and participant consent

Ethical clearance and approval were received from the Institutional Review Board (IRB) of the University of Gondar with Ref. No. VP/RCS/05/551/2020. Before having them complete the questionnaire, study participants were first given a verbal explanation of the study purpose. If they were agreeable, then participants gave their written informed consent. For the individuals >18 years, written informed assent was obtained from their parents. Confidentiality was maintained by omitting their personal identification. Participants were not forced to participate or received any monetary incentive, and it was solely voluntary based.

## Result

### Socio-demographic and family-related characteristics of young people with the substance use

Of the total, 296 (79.57%), 199 (53.49%), 127 (34.14%), and 60 (16.13%) participants were consuming alcohol, Khat, tobacco/cigarette products, and other substances (shisha, inhalants, and drugs), respectively. Notably 274 (73.66%) were men, 316 (84.95%) were orthodox Christians, 184 (49.46%) attended secondary school, and 310 (83.33%) were single in their marital status. More than half of the respondents (58.3%) were students, 266 (71.51%) were living with their family, and 108 (29.04%) lost beloved family members recently. The majority of those (59.67) were urban and semi-urban residents. The mean age of young people with the substance use was 20.51 (2.61 SD) years (Table 1).

**Table 1:** Socio-demographic and family-related characteristics of the participants in Northwest Ethiopia (*n* = 372).

**Variables**	**Categories**	**Frequency**	**Percentage (%)**
District	Gondar	45	12.10
	Alefa	79	33.33
	Chilga	62	16.67
	East Belesa	54	14.52
	Tach Armachiho	18	4.84
	Gondar Zuria	114	30.65
Sex	Male	274	73.66
	Female	98	26.34
Age	15-19 years	152	40.86
	20-25 years	220	59.14
Religion	Orthodox	316	84.95
	Muslim	48	12.90
	Others	8	2.15
Marital status	Single	310	83.33
	Married	55	14.78
	Divorced/separated	7	1.89
Living with	Alone	95	25.54
	With family	266	71.51
	Others	11	2.96
Residency	Urban	222	59.68
	Rural	150	40.32
Educational level	Cannot read and write	31	8.33
	Primary education	79	21.24
	Secondary education	184	49.46
	Collage and above	78	20.97
Occupation	Government employed	30	8.06
	Merchant	55	14.78
	Student	217	58.33
	Farmer	53	14.25
	Jobless	17	4.57
Biological parents alive	Yes	308	82.80
	No	64	17.20
Recent loss of families	Yes	109	29.30
	No	263	70.70
DASS stress scale	Normal	154	41.40
	Mild	59	15.86
	Moderate	90	24.19
	Severe	69	18.55
Perceived social support	Low	43	11.56
	Moderate	199	53.49
	High	130	34.95

### Prevalence of psychotic symptoms among the youth who uses substances

Among the substance use participants, the prevalence of psychotic symptoms was 24.19% with a 95% confidence interval between 20.13% and 28.83%. More than one-fourth (27.4%) of alcohol users, 28.6% of Khat chewers, 31.5% of tobacco/cigarette smokers, and 33.3% of other substances (shisha, inhalants, and drugs) users had psychotic symptoms. In total, 66 (73.33%) of the participants who had psychotic symptoms were male subjects. More than half of the respondents (52.22%) who had psychotic symptoms had moderate perceived social support from family, friends, and significant others.

### Factors associated with psychotic symptoms

As indicated in Table 2, the bi-variable binary logistic regression showed that sex, age, marital status, residence, loss of loved ones, perceived social support, and psychological stress fulfilled the *p*-value of < 0.25. In the multivariate logistic model, married individuals, the recent loss of loved ones, poor perceived social support, and severe psychological stress were significantly associated with psychotic symptoms at a *p*-value of < 0.05.

**Table 2:** Bi-variable and multivariable binary logistic regression of factors associated with psychotic symptoms among the youth population with substance use in the Central Gondar Zone, Northwest Ethiopia, 2021 (*n* = 372).

**Variables**	**Having psychotic symptoms**		**COR (95% CI)**	**AOR (95% CI)**
	**No (** ***n*** **)**	**Yes (** ***n*** **)**		
**Sex**				
Male	208	66	1	1
Female	74	24	1.02 (0.84, 1.36)	1.28 (0.71, 2.27)
**Age in years**				
15-19	120	32	1	1
20-25	162	58	1.34 (0.88, 2.14)	0.91 (0.52, 1.58)
**Marital status**				
Married	36	19	1.83 (1.02, 3.34)	1.87 (1.06, 3.48)^*^
Unmarried and/ divorced	246	71	1	1
**Residence**				
Rural	119	31	1	1
Urban	163	59	1.39 (0.85, 2.26)	1.46 (0.86, 2.47)
**Having recent loss**				
No	209	54	1	1
Yes	73	36	1.91 (1.16, 3.14)	1.97 (1.10, 3.18)^**^
**Perceived Social support**				
Low	28	15	1.95 (1.24, 3.16)	1.61 (1.11, 3.02)^*^
Moderate	152	47	1.13 (0.66, 1.92)	0.90 (0.51, 1.54)
High	102	28	1	1
**Psychological stress**				
Normal	128	26	1	1
Mild	45	14	1.53 (0.74, 3.19)	1.50 (0.70, 3.21)
Moderate	68	22	1.59 (0.84, 3.02)	1.51 (0.75, 3.00)
Severe	41	28	3.36 (1.77, 6.37)	3.23 (1.64, 6.54)^**^

COR, crude odds ratio; AOR, adjusted odds ratio; CI, confidence interval; 1 = reference category; ^*^p < 0.05; ^**^p ≤ 0.001; Hosmer–Lemeshow goodness-of-fit test = (p-value = 0.17).

The odds of having psychotic symptoms among young married people with psychoactive substance use were 1.87 times [AOR = 1.87; 95% CI: 1.06, 3.48] higher as compared to unmarried/divorced young people. The odds of psychotic symptoms were two times [AOR = 1.97; 95% CI; 1.10, 3.18] higher among the youth population who had a recent loss of loved ones than those who had no recent loss. Individuals who had low perceived social support had 1.61 times [AOR = 1.61; 95% CI: 1.11, 3.02] higher odds of psychotic symptoms compared with individuals who had high perceived social support. The participants who had severe psychological stress were three times [AOR = 3.23, 95% CI: 1.64, 6.54] more likely to develop psychotic symptoms than those with no psychological stress.

## Discussion

The prevalence of psychotic symptoms among the youth population with psychoactive substance use in Northwest Ethiopia was 24.24% with a 95% CI between 20.13 and 28.83%. The finding of this study was consistent with previous studies done in Petersburg, Russia (21%), and Munich, German (23.8%) (33, 34). The finding of this study is higher than other studies conducted in Tanzania (3.9%) (35), Britain (5.5%) (36), and Spain (11.2%) (37). The discrepancy might be due to the variation in the screening tools used, the study design, sample size, participants' characteristics, and socioeconomic status. A longitudinal study on adult participants employed in Britain and Spain showed that 5.5% and 11.2% of the participants had newly occurring psychotic symptoms assessed using the psychosis screening questionnaire (36, 37), and the participants in Tanzania's study were urban residents, of whom 3.9% have psychotic symptoms (35). The current study was a population cross-sectional study on young people with a high sample of participants than the previous studies. In addition, the current study used a sample drawn from youth populations aged between 15 and 25 years while the previous studies included ages beyond 24. This may be attributed to the young part of the population with psychoactive substances who are highly susceptible to the negative mental health impacts of substance use. Discrepancies among young people with psychoactive substance use having comorbid psychotic symptoms could be associated with social-economic and health policy differences.

However, the finding of the current study is lower than the longitudinal study conducted in Canada, from 2008 to 2015 among people living in precarious housing or homelessness in which 79.3% of the participants had psychotic symptoms (38). This study's result was also lower than another study in which the magnitude of specific psychotic symptoms such as persecutory delusions and auditory hallucinations were 77.4 and 44.6%, respectively, among amphetamine users (39). The reason for the high difference in the magnitude of psychotic symptoms might be due to the difference in the characteristics of study populations. For instance, in a study performed in Canada, one-third of the participants grew up in foster care or were adopted (38), and the other study's participants were adults with amphetamine use. These factors are frequently associated with psychotic symptoms. This study had a relatively very short period of study than those studies performed in previous times. The age of participants in the current study was limited to be in between 15 and 25 years that is very different from the rest of the studies that incorporated children, adults, and older adults. In addition, the current study has addressed all kinds of psychoactive substances that may contribute to the lower prevalence of psychotic symptoms. Unlike some other studies performed on psychotic symptoms related to psychoactive substance use, this study did not specify participants of special populations such as participants with general medical conditions, epileptic patients, poly-victimized (40–42), or prior history of psychosis (38).

This study showed that psychotic symptoms among the youth population with psychoactive substance use were significantly associated with married individuals in marital status, the recent loss of loved ones, low perceived social support, and severe psychological stress. Psychotic symptoms of comorbidity with psychoactive substance use were significantly associated with being married. This finding was inconsistent with those of previous studies (35, 43, 44), because these studies reported that being married is a protective factor for the development of psychotic symptoms and those who were not married, divorced, and/or widowed have higher odds of psychotic symptoms. A possible justification could be the young population who were married might use psychoactive substances for a longer time with a higher amount than the counter. Psychoactive substance use for a longer time is a known contributor to the high incidence of developing psychotic symptoms among the young population (45).

In comparison to individuals who had no recent bereavement, young people who use psychoactive substances and have recently lost loved ones are twice as likely to exhibit psychotic symptoms. Young people with recent losses and a history of substance abuse reported more severe psychotic symptoms. It is obvious that experiencing a recent loss of a loved one triggers psychological effects, such as disruptions in perception and cognition (46). Low perceived social support was also significantly associated with psychotic symptoms among the youth population with psychoactive substance use. The finding is in line with other studies from Tanzania (35) and Great Britain (44), which found that a strong social network lowers the risk of psychotic symptoms and prevents young people from psychiatric disorders caused by the use of psychoactive substances. The lack of social support may cause psychological anguish, a sense of isolation and powerlessness, and a perception of disadvantage. Then, individuals might experience depression symptoms, mental abnormalities, impairment of perception, and sleep disruptions (19).

Severe psychological distress among young people with substance use was strongly associated with psychotic symptoms compared to those with no psychological distress. Some studies support the finding of this study (47, 48). This may be because people who are stressed and experiencing negative emotions turn to psychoactive drugs and alcohol to deal with their problems and find relief. Psychiatric problems originate from the combination of psychological stress and psychoactive substances. Due to their low resilience, the juvenile population that uses psychoactive substances and is in psychological distress is more prone to experiencing psychotic-like experiences, including delusion and hallucination (49). Furthermore, it may be because of the behavioral correlation between using a certain psychoactive substance and psychological suffering, and it is possible that psychotic symptoms force people to utilize drugs as a coping mechanism for their negative emotions.

### Strengths and limitations of the study

In the current study, psychotic symptoms were measured using a standardized tool that is validated for both high- and low-income countries. The study was community-based, and this may help to generalize the results to the population. The study also used appropriate statistical analysis to estimate the effect of different independent variables on the dependent variable. The limitations of this study include the cross-sectional nature of the study that raises the issue of causal–effect relationships. It was not possible to disentangle associated factors for psychotic symptoms whether they were prior to the use of the psychoactive substances or due to independent factors. Recall or social desirability response bias might also be obvious due to the nature of the study.

## Conclusion

The prevalence of psychotic symptoms related to psychoactive substances among the youth population in Northwest Ethiopia was high. Individuals who are married, have recently lost their loved ones, have low perceived social support, and have severe psychological distress were the factors significantly associated with psychotic symptoms. For young people who have recently lost loved ones, have low social support, and have psychological distress, it is essential that policymakers provide psychosocial support for the purpose to increase their resilience and reduce the occurrence of Psychotic like symptoms. Again, special interventions targeting risky youngsters like those living with these psychological instabilities are also needed to be intervened in the psychotic-like experiences concurrent with psychoactive substance use at an early age. It is better to undertake a longitudinal study with multiple repeated measures of psychotic symptoms and risk factors over many years to identify the possibility of problems due to the issues related to causal–effect relationships between the dependent and independent variables.

## Data availability statement

The original contributions presented in the study are included in the article/supplementary material, further inquiries can be directed to the corresponding author.

## Ethics statement

The studies involving human participants were reviewed and approved by the Institutional Review Board (IRB) of the University of Gondar. The patients/participants provided their written informed consent to participate in this study.

## Author contributions

All authors made a significant contribution to the work reported, whether that is in the conception, study design, execution and acquisition of data, analysis, interpretation, took part in drafting, revising, or critically reviewing the article, gave final approval of the version to be published, and agreed on the journal to which the article has been submitted.
